# Reversible Isolated Opsoclonus in a Patient With Gastric Cancer and Antibodies to Glutamic Acid Decarboxylase and Myelin Oligodendrocyte Glycoprotein

**DOI:** 10.7759/cureus.101880

**Published:** 2026-01-19

**Authors:** Alekhya Bommireddipalli, Arin Boghoz, Aengela Jihyoun Kim, Jennifer Shieh, Michael Yco, Natalie Karadanaian, Antonio K Liu

**Affiliations:** 1 Internal Medicine, Adventist Health White Memorial, Los Angeles, USA; 2 Family Medicine, Adventist Health White Memorial, Los Angeles, USA; 3 Neurology, Adventist Health White Memorial, Los Angeles, USA; 4 Neurology, School of Medicine, Loma Linda University, Loma Linda, USA

**Keywords:** gastric cancer, glutamic acid decarboxylase (gad) antibodies, myelin oligodendrocyte glycoprotein (mog) antibodies, non-neoplastic neurological abnormality, opsoclonus

## Abstract

Opsoclonus and myoclonus are known paraneoplastic manifestations of malignancy. The most common antibodies found are anti-neuronal antibodies (Hu, Ri, and Ma). The most common affiliated malignancies are small-cell lung cancers, breast cancers, or gynecological malignancies. Gastric cancer is uncommon. Other antibodies have been cited, but are rare. Isolated opsoclonus without significant myoclonus is also rare. While both glutamic acid decarboxylase (GAD) and myelin oligodendrocyte glycoprotein (MOG) antibodies have individually been reported to be associated with opsoclonus, reports of a combination of these two in a patient with gastric cancer with isolated reversible opsoclonus are scarce. In this case, we follow a patient who presented as such and demonstrated significant symptomatic improvement after the administration of intravenous immunoglobulin.

## Introduction

Opsoclonus is an involuntary, rapid, multidirectional, and conjugate abnormal ocular movement. It can be a manifestation of paraneoplastic, autoimmune, postinfectious, and toxic-metabolic diseases. Opsoclonus is frequently associated with myoclonus, such as in opsoclonus-myoclonus syndrome (OMS). Isolated opsoclonus is rare. In normal physiology, saccade commands are transmitted from the cerebrum to the brainstem, where various brainstem-cerebellar circuitries are involved in the smooth pursuit of extraocular movement. The fastigial nucleus, vestibulocerebellar tracts, and omnipause neurons play key roles in eye movements and saccadic control. Damage to these structures disrupts saccade control, lessens the suppression of saccades, and results in involuntary saccades [1].

OMS is a rare nervous system disorder featuring characteristic abnormal eye movement and myoclonus, usually paraneoplastic in nature with probable autoimmune etiology. Besides central ocular motor dysfunction, OMS is characterized by clinical features, such as involuntary multifocal myoclonus, cerebellar ataxia, and uncoordinated voluntary movements. In children, OMS is predominantly related to neuroblastoma. While in adults, it can be evident in small-cell lung cancer, breast cancer, ovarian cancer, melanoma, sarcoma, and non-Hodgkin lymphoma. Most often, OMS presents positive anti-neuronal antibodies in cerebrospinal fluid (CSF) and serum analysis, specifically, anti-Ri, Hu, Yo, and Ma. Many other autoimmune antibodies have been reported, but they are infrequent [2]. Moreover, many cases of paraneoplastic OMS are anti-neuronal antibody negative [2]. However, a full assessment and workup, including specific antibody marker testing of serum and CSF, should still be performed for an accurate diagnosis. The exact pathophysiology is only postulated, but certain researchers suggest that the affected cerebellar nuclei are disinhibited due to consistent IgG and B cell-mediated autoimmunity against the synthesis of the inhibitory neurotransmitter gamma-aminobutyric acid (GABA), which further prevents GABA inhibitory signals from coordinating full inhibition of the affected cerebellar neurons, resulting in incomplete suppression of unintended extraocular movement [2].

Glutamic acid decarboxylase (GAD) is an enzyme that catalyzes the decarboxylation of glutamate to gamma-aminobutyric acid (GABA), a main inhibitory neurotransmitter within the central nervous system. GAD antibodies have been associated with multiple neurological syndromes, including (but not limited to) stiff-person syndrome, cerebellar ataxia, limbic encephalitis, refractory status epilepticus, and nystagmus [3]. Reduced GABAergic transmission, which causes neuronal excitability, is considered to be a possible mechanism. Many patients suffer from overlapping syndromes as well. The reasons for the clinical heterogeneity among GAD-antibody-associated syndromes remain unclear; there may be differences in the susceptibility of GABAergic neurons to anti-GAD antibodies or to other, as yet unidentified, autoantibodies [3]. It remains uncertain what causes the presence of these antibodies, why they persist, and whether they are disease markers or have pathogenic potential [3]. The pathogenic role of GAD antibody is still debated, and the diagnosis relies on the detection of GAD antibodies in serum and/or cerebrospinal fluid. Due to the relative rarity of these syndromes, treatment schemes and predictors of response are not well reported or studied. Pathologies associated with GAD antibody can be both paraneoplastic and non-paraneoplastic in nature.

Myelin oligodendrocyte glycoprotein (MOG) has emerged as a target of demyelinating autoantibodies in a wide range of acquired inflammatory demyelinating diseases of the central nervous system (CNS), such as multiple sclerosis (MS) and other disorders, including neuromyelitis optica (NMO), acute disseminated encephalomyelitis (ADEM), Marburg disease, Balo concentric sclerosis, and Schilder disease [4]. Evidence exists that MOG might act as a cell adhesion molecule, a regulator of microtubule stability, and a mediator of interactions between myelin and the immune system [4]. Cell-based immunoassays using MOG expressed in mammalian cells have demonstrated the presence of high-titer MOG antibodies in pediatric patients with ADEM, MS, aquaporin-4-seronegative NMO, isolated optic neuritis, or transverse myelitis. However, high-titer MOG antibodies are rare in adults with these disorders [4]. A limited number of reports have described MOG’s association with OMS; however, descriptions of isolated opsoclonus with MOG antibody in a paraneoplastic context remain scarce, as discussed in subsequent sections.

We report a patient with metastatic gastric cancer and isolated, reversible opsoclonus. Considering that there is no universally established relationship between opsoclonus and gastric cancer, this is deemed paraneoplastic. With two uncommon antibodies positive simultaneously, our case is rare and deserves reporting to raise awareness among clinicians.

## Case presentation

A 71-year-old male with metastatic gastric adenocarcinoma receiving chemotherapy presented to the hospital for one week of visual disturbances. He was diagnosed with stage 4 disease and had undergone multiple sessions of chemotherapy. On this admission, he subjectively reported progressive "object drifting" and "jerking of the vision" with upward and lateral gaze at first. As symptoms progressed, it began to occur even when he looked straight ahead. He remained asymptomatic only when looking downward. Visual acuity was not affected, and he denied diplopia. He was unable to grab objects. At some point, he could not keep his eyes open, as he felt dizzy and extremely nauseated. His ability to walk became severely impaired, as he could only open his eyes briefly to find a wall to hold in order to travel short distances before needing to scan his environment again. He could walk normally with his eyes closed. He denied a change in mentation. On examination, the patient appeared cachectic and highly uncomfortable, with eyes shut tightly due to feeling nauseated. His vital signs were stable. He was coherent and could provide a detailed history. During the eye exam, we could only observe his eyes for a few seconds each time, as he was unable to keep them open before he became too dizzy, nauseated, or uncomfortable. Both eyes moved in unison and in a conjugate manner. He could see, but described everything as "flying around" at high speed, back and forth. When he looked straight ahead, he exhibited multidirectional conjugate saccades without a slow phase. Upward gaze exacerbated these jerks, both in frequency and duration. With lateral gaze, horizontal saccades became predominant. These movements were too fast to allow pupil examination. The rest of the cranial nerve examination was non-revealing. He had preserved strength. He could not perform a finger-to-nose examination with his eyes open because he could neither track nor accommodate appropriately. However, after closing his eyes, he could estimate the location of the tester’s finger and complete the test. He had no obvious dysmetria or dysdiadochokinesia.

**Figure 1:**
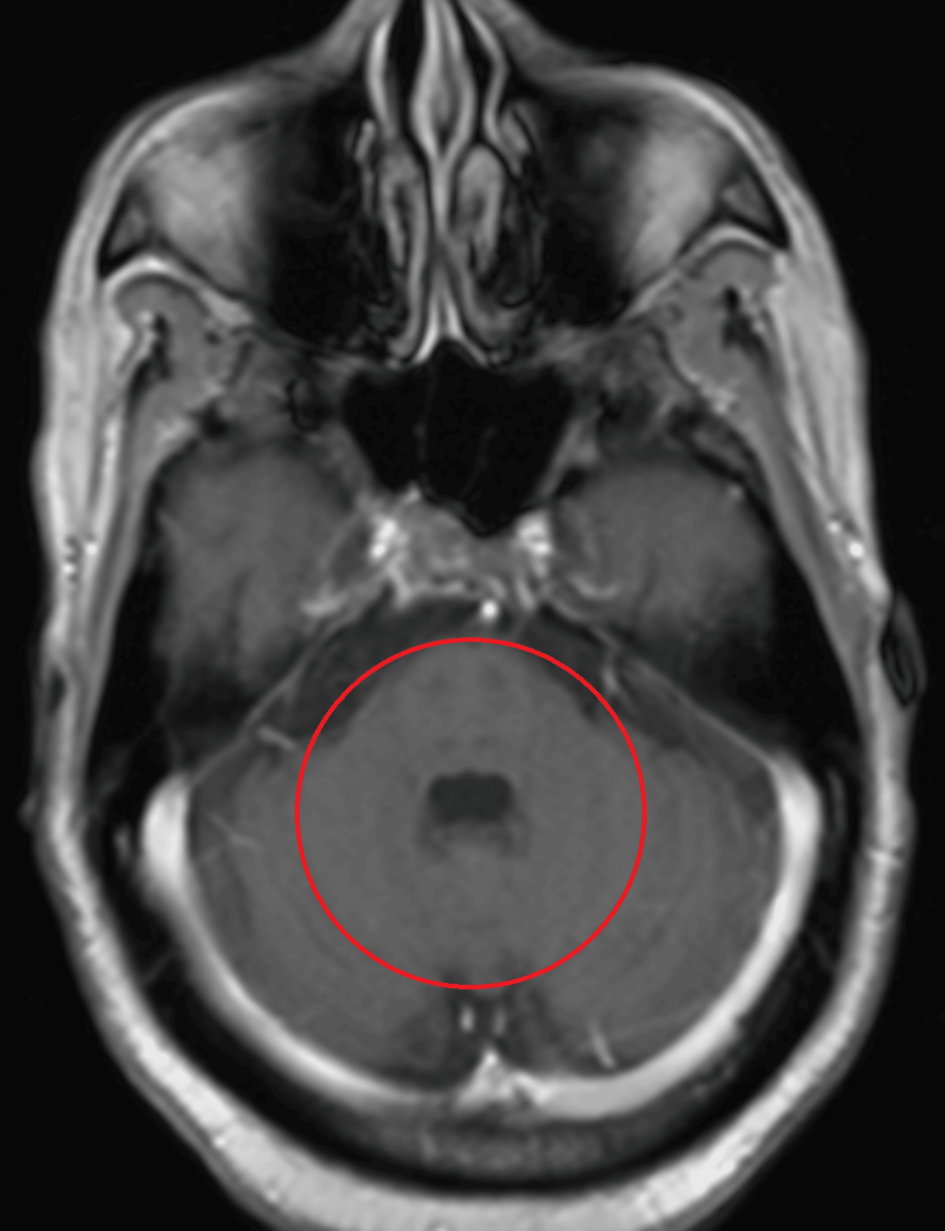
T1 postcontrast image of brain MRI showing no enhancing lesion in pons or cerebellum. The red circle shows the area of interest.

**Figure 2:**
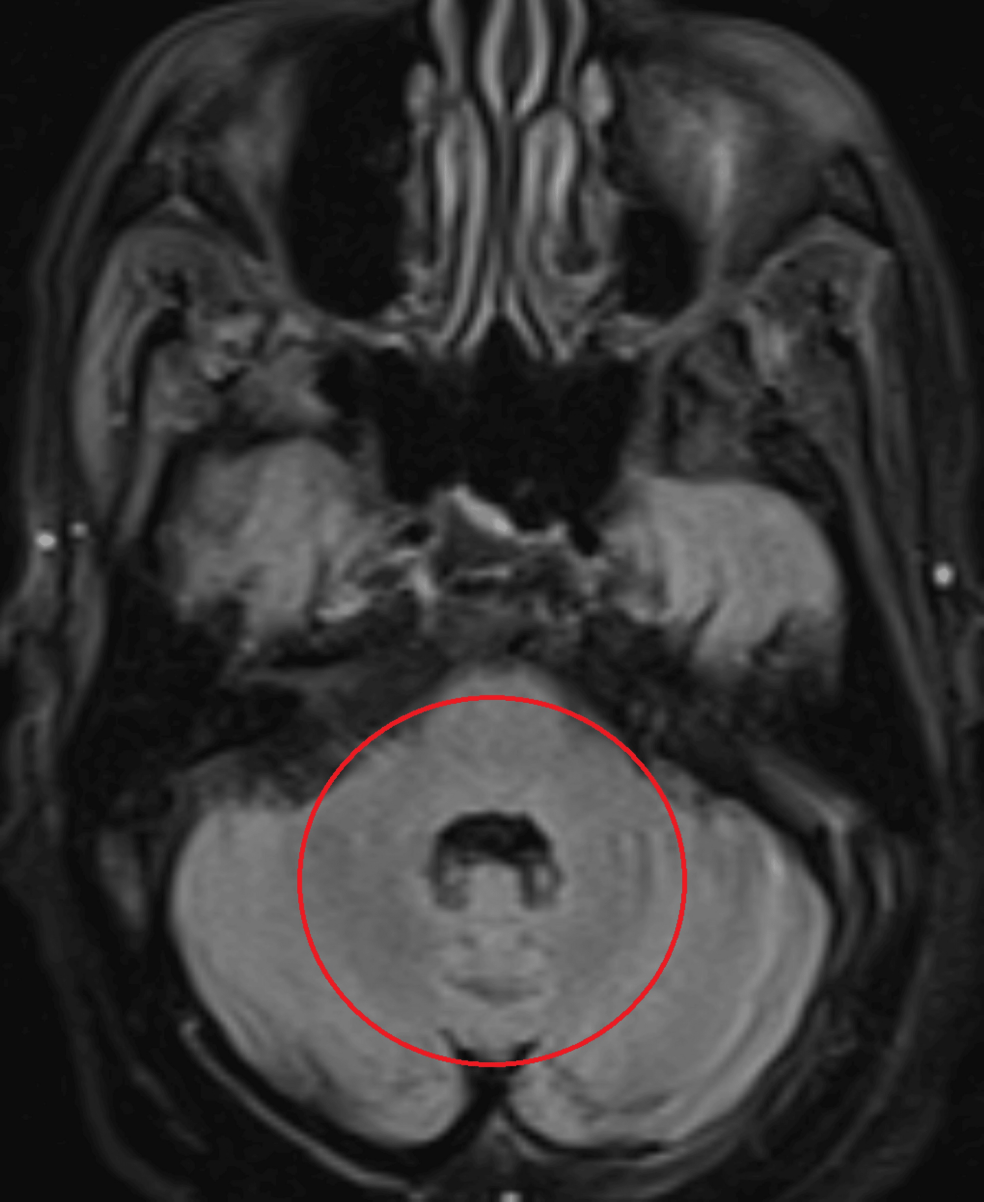
FLAIR sequence of brain MRI showing no abnormality in pons or cerebellum. The red circle shows the area of interest. FLAIR: fluid-attenuated inversion recovery

**Figure 3:**
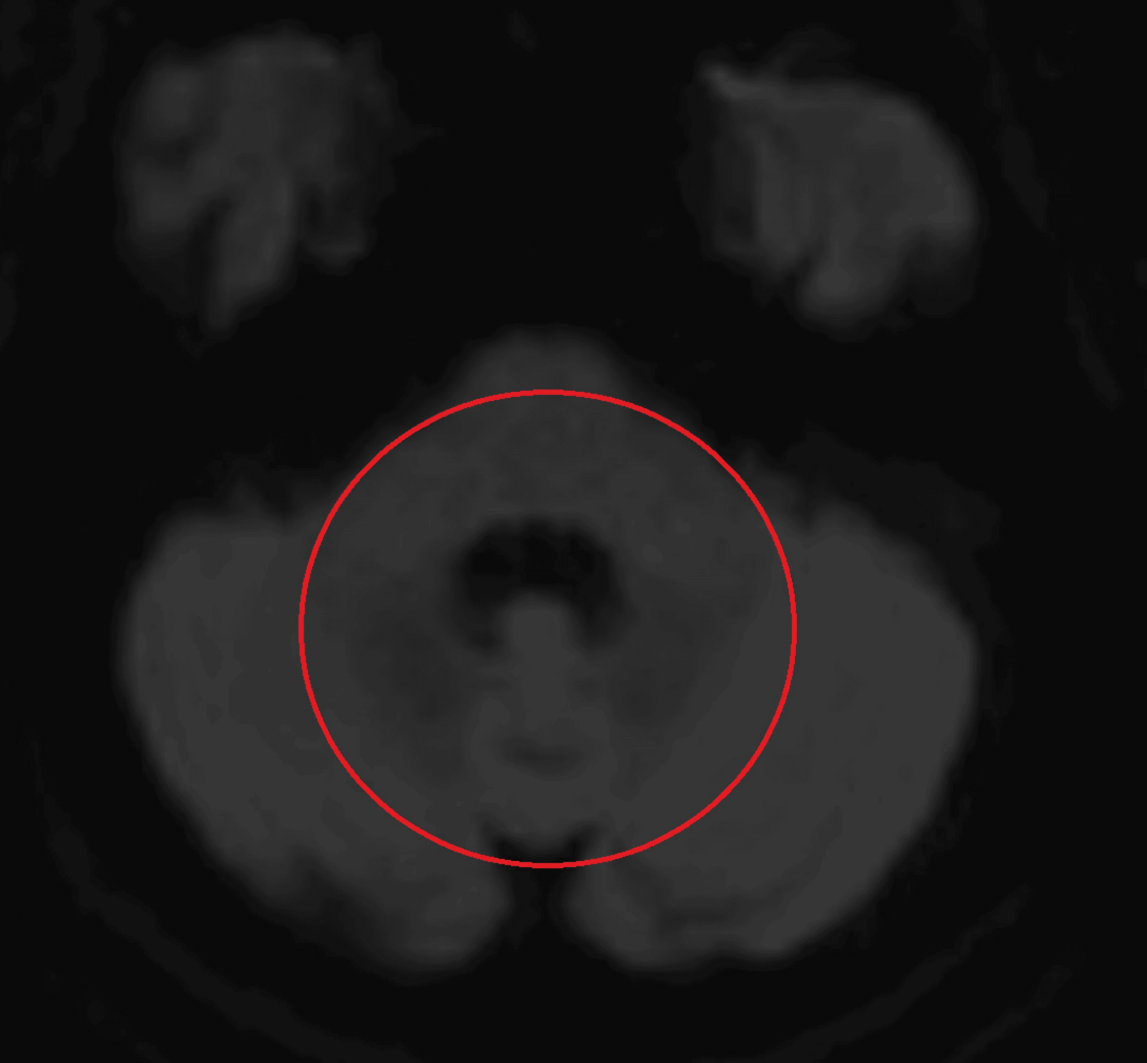
DWI sequence of brain MRI showing no restricted diffusion abnormality in pons or cerebellum. The red circle shows the area of interest. DWI: diffusion-weighted imaging

MRI of the head, with and without contrast, was negative for stroke or masses (Figures 1-3). General laboratory studies, chemistry, and blood count were all non-revealing. His B12 was 1500 pg/mL. An autoimmune neurologic disease antibody panel was sent. Among 23 antibodies tested, GAD, MOG, and voltage-gated potassium channel (VGKC) antibodies were positive (Table 1). Anti-Hu, Ri, Yo, and Ma-2 antibodies were negative. Although VGKC antibody was borderline elevated, leucine-rich glioma-inactivated 1 (LGI1) and contactin-associated protein-like 2 (CASPR2) antibodies were all negative. Patient declined cerebral spinal fluid (CSF) analysis with lumbar puncture. With paraneoplastic etiology highly suspected, five-day courses of both intravenous immunoglobulin (IVIG) 400 mg/kg/day as well as methylprednisolone IV 1000 mg/day were administered. The patient experienced improvement of symptoms by day four of Solu-Medrol. His visual disturbance steadily improved and eventually completely resolved. Five days after IVIG and steroid completion, all of his symptoms had resolved. No further maintenance immunomodulation therapy was needed.

**Table 1:** Pertinent positive and negative laboratory findings. MOG: myelin oligodendrocyte glycoprotein; NMO: neuromyelitis optica; AQP4: aquaporin 4; LGI1: leucine-rich glioma-inactivated 1; CASPR2: contactin-associated protein-like 2

Parameters	Value	Reference range
B12	>1500 pg/mL	280-980 pg/mL
Neuronal antibody (Hu)	Negative	Negative
Neuronal antibody (Ri)	Negative	Negative
Neuronal antibody (Yo)	Negative	Negative
Neuronal nuclear antibody (TR/DNER)	Negative	Negative
Neuronal antibody (amphiphysin)	Negative	Negative
Purkinje cell/neuronal nuclear IgG	Not detected	Not detected
P/Q-type calcium channel antibody	0.0	0.0-24.5 pmol/L
NMO/AQP4 Ab IgG	<1:10	<1:10
MOG antibody	Detected	<1:10
Glutamic acid decarboxylase antibody	>250.0 IU/mL	0.0-5.0 IU/mL
Voltage-gated potassium channel antibody	43 pmol/L	Intermediate 32-87 pmol/L
LGI1 antibody	<1:10	<1:10
CASPR2 antibody	<1:10	<1:10

Unfortunately, the patient was lost to direct neurological follow-up. We learned from colleagues, unfortunately, that his cancer has progressed, and he developed generalized body weakness and became bedridden. There was no recurrence of visual disturbance to our knowledge.

## Discussion

Upon first impression, opsoclonus is often associated with neurological disease. In our patient with gastric cancer, brain imaging and further clinical findings were not consistent with a discrete neurological etiology. Malignancies, however, have a variety of paraneoplastic phenomena that are still being established and understood. The presence of serum GAD and MOG, as well as low-level voltage-gated potassium channel (VGKC) antibodies, points towards an autoimmune etiology. He demonstrated continuous multidirectional conjugate saccades without a slow phase; there was no nystagmus at any time. He did not have myoclonus, dysmetria, or dysdiadochokinesia. His rapid response to intravenous immunoglobulin (IVIG) and methylprednisolone also suggests an autoimmune cause. However, there are some limitations to this case. The lack of CSF studies means we were unable to confirm the presence of GAD or MOG antibodies in CSF and quantify the MOG antibodies. Moreover, had CSF analysis been available, we would have been able to evaluate for the presence of carcinomatous meningitis. However, the lack of involvement of cranial nerves, headache, and altered mentation suggests that there was probably no infiltrative disease of the meninges. CSF may not have contributed to this patient’s pathology. In addition, lack of clinical follow-up and the inability to follow MOG/GAD levels render us unable to confirm the absence of relapse and the clearance of the antibodies.

Isolated opsoclonus is extremely rare in the context of GAD autoimmunity, and the few reported cases involve opsoclonus-myoclonus-ataxia syndrome rather than isolated opsoclonus. The literature supports the notion that GAD antibody-associated neurological syndromes encompass a broad spectrum, including stiff-person syndrome, cerebellar ataxia, and limbic encephalitis, but isolated opsoclonus is not well documented. Many cases of opsoclonus-myoclonus syndrome (OMS) are idiopathic in nature with no associated malignancies [5-7]. GAD-associated OMS is found in paraneoplastic settings, such as lung and thymic malignancies, rather than gastric cancers [8,9]. One case report describes both opsoclonus and downbeat nystagmus in a patient with high anti-GAD titers, suggesting that GAD autoimmunity can produce diverse oculomotor disturbances, but isolated opsoclonus remains underreported [6]. These findings support the idea that GAD autoimmunity produces a broader spectrum of oculomotor disturbances than previously recognized. Isolated opsoclonus, while rare, may represent an underreported manifestation, warranting further investigation. Many reports found that symptomatic treatment with baclofen, diazepam, and immunomodulation via plasma exchange or intravenous immunoglobulin was beneficial [7,8]. Our patient received IVIG along with Solu-Medrol, with improvement of symptoms.

MOG, besides playing a central role in MOG antibody-associated disease with optic neuritis and transverse myelitis, has also been documented to be involved in OMS. Isolated OMS is reported in a child, and demyelination is involved, leading scholars to add it to the forever-expanding spectrum of MOGAD [10]. In other situations, they are neither neoplastic nor demyelinating in nature. One case concerns COVID [11], and another concerns postpartum (which involves significant immune modulation and increased susceptibility to autoimmune disease) [12]. This underscores the MOG antibody’s role in targeting the saccade circuitry even before demyelination sets in, or independent of demyelination completely. MOG affiliation with malignancy is extremely rare, with only a few cases in existence [13]. It was found that 3.6% of 445 patients with MOGAD had developed neoplasms within two years; after a general literature search, reports of opsoclonus as part of MOG-associated neoplastic presentation are scarce. Taken together, MOG antibody may play a paraneoplastic role in brainstem circuitries that affect saccades, independent of demyelination.

OMS is found in both idiopathic and paraneoplastic syndromes. As a paraneoplastic condition, it is usually associated with breast cancer and small-cell lung cancer. One study found OMS in 10 individuals with lung cancer, as well as two out of 14 breast cancer patients total. Reports describing OMS in association with gastric cancer are limited. In those instances, anti-Hu, anti-amphiphysin, and anti-Ri were detected [14]. A larger-scale study found 63 out of 137 patients with OMS had some sort of malignancy. The only antibody detected and reported was Anti-Ri [15]. Lung and breast cancers, again, were the main cancers detected, with no instance of gastric cancer. One case did report opsoclonus with no mention of myoclonus in a patient with gastric cancer. However, it is different from our case since the antibody identified was anti-Ma2, and the patient also had limbic encephalitis [16]. Other common antibodies found are anti-Hu and anti-Yo [17,18]. Isolated opsoclonus is infrequently described, and reports involving GAD antibodies in the setting of gastric cancer are scarce.

Gastric cancer is not the most common tumor associated with paraneoplastic manifestations. One case reported a presumed autoimmune paraneoplastic encephalitis, though no antibody was identified [19]. A case of cerebellar degeneration without opsoclonus, associated with anti-Ri antibodies, was found in a patient with neuroendocrine gastric carcinoma [20]. Another patient with gastric adenocarcinoma and ataxia was found to have anti-Yo antibodies. The immune response against the Purkinje cells likely caused cerebellar degeneration, leading to the ataxia [21]. There was also an interesting report of reversible cerebellar atrophy in a patient with gastric adenocarcinoma; no opsoclonus was observed, and the antibody detected was amphiphysin [22]. A case of MOG antibodies in a patient with a gastric carcinoid tumor was reported, but the patient had classic neuromyelitis optica, and no opsoclonus was noted [23]. No association between GAD antibodies and gastric cancer was identified in our literature search.

The possible mechanism underlying our observation is the dysfunction of omnipause neurons and fastigial nucleus in the pons, which normally suppress inappropriate saccadic activity, causing excessive saccadic eye movement, which is the basis of opsoclonus. Anti-Hu and anti-Ri antibodies are the most commonly identified paraneoplastic anti-neuronal antibodies, usually associated with lung and breast cancer [1]. It is important to understand that anti-Hu and anti-Ri antibodies attack nuclear and cytoplasmic neuronal antigens. GAD, on the other hand, is a synaptic protein involved in the synthesis, packaging, and release of gamma-aminobutyric acid (GABA) [24]. Anti-GAD antibodies suppress GABA release at the terminal of inhibitory neurons [25]. Suppression of GABA, also known as paucity in GABA, has been implicated in the dysfunction of these brainstem circuitries [26,27]. Likewise, such reduction in inhibition to Purkinje cells was observed in MOG related encephalomyelitis; MOG antibody can suppress GABAergic signaling as observed in multiple sclerosis [28]. Such observation has therapeutic implications. Antibodies against cell-surface or synaptic proteins (like GAD) can potentially be reversed by immunotherapy more so than cytoplasmic elements [24].

In summary, while numerous combinations of cancers, antibodies, and symptoms are possible, the most common antibodies identified in association with opsoclonus are Hu, Ri, and Ma. Isolated reversible opsoclonus with gastric cancer, positive GAD and MOG antibodies rarely occur, to our knowledge. We are hereby reporting these uncommon observations in the hope of broadening the disease spectrum [29].

## Conclusions

Many paraneoplastic presentations have well-known established associations with certain cancers, such as Lambert-Eaton, hypercalcemia of malignancy, and symptoms of inappropriate anti-diuretic hormone secretion. While OMS is typically thought to associate itself with small-cell lung cancer and breast cancers, we present an atypical scenario in which a patient with metastatic gastric cancer demonstrates an isolated opsoclonus that was able to be treated with intravenous immunoglobulin. Further testing revealed positive antibodies to both GAD and MOG, both of which have not had a previously established relationship with either isolated opsoclonus or gastric cancer. Our case highlights that paraneoplastic syndromes may manifest in conjunction with different malignancies and in association with different antibodies. It reinforces the importance of aggressive diagnostic assessment and prompt treatment of suspected paraneoplastic phenomena despite initial clinical impression in order to maximize quality outcomes for patients.
